# Has the OSCE Met Its Final Demise? Rebalancing Clinical Assessment Approaches in the Peri-Pandemic World

**DOI:** 10.3389/fmed.2022.825502

**Published:** 2022-02-21

**Authors:** Bunmi S. Malau-Aduli, Karina Jones, Shannon Saad, Cassandra Richmond

**Affiliations:** ^1^College of Medicine and Dentistry, James Cook University, Townsville, QLD, Australia; ^2^School of Medicine, Notre Dame University, Sydney, NSW, Australia

**Keywords:** OSCE (Objective Structured Clinical Examination), pandemic (COVID-19), clinical assessment, medical education, qualitative study

## Abstract

The Objective Structured Clinical Examination (OSCE) has been traditionally viewed as a highly valued tool for assessing clinical competence in health professions education. However, as the OSCE typically consists of a large-scale, face-to-face assessment activity, it has been variably criticized over recent years due to the extensive resourcing and relative expense required for delivery. Importantly, due to COVID-pandemic conditions and necessary health guidelines in 2020 and 2021, logistical issues inherent with OSCE delivery were exacerbated for many institutions across the globe. As a result, alternative clinical assessment strategies were employed to gather assessment datapoints to guide decision-making regarding student progression. Now, as communities learn to “live with COVID”, health professions educators have the opportunity to consider what weight should be placed on the OSCE as a tool for clinical assessment in the peri-pandemic world. In order to elucidate this timely clinical assessment issue, this qualitative study utilized focus group discussions to explore the perceptions of 23 clinical assessment stakeholders (examiners, students, simulated patients and administrators) in relation to the future role of the traditional OSCE. Thematic analysis of the FG transcripts revealed four major themes in relation to participants' views on the future of the OSCE vis-a-vis other clinical assessments in this peri-pandemic climate. The identified themes are (a) enduring value of the OSCE; (b) OSCE tensions; (c) educational impact; and (d) the importance of programs of assessment. It is clear that the OSCE continues to play a role in clinical assessments due to its perceived fairness, standardization and ability to yield robust results. However, recent experiences have resulted in a diminishing and refining of its role alongside workplace-based assessments in the new, peri-pandemic programs of assessment. Future programs of assessment should consider the strategic positioning of the OSCE within the context of utilizing a range of tools when determining students' clinical competence.

## Introduction

Since its initial introduction as a mode of assessment for medical students in the 1970s (1), the Objective Structured Clinical Examination (OSCE) has become increasingly favored as the method of clinical assessment in undergraduate and postgraduate health professions education (2, 3). The OSCE assesses clinical competence, based on objective testing through direct observation of student performances in simulated clinical scenarios. Published research about the OSCE from its inception has reported it to be a reliable, valid and objective instrument for assessment (2–4). The benefits of the OSCE include its standardized approach to the assessment of clinical competence in differing cultural and geographical contexts; and its ability to assess a wide range of learning outcomes in varying specialties and disciplines for both formative and summative purposes at all phases of health professions education, from the early years of the undergraduate curriculum to postgraduate specialty training (5, 6).

Despite the general acceptance of the OSCE as the preferred tool for clinical assessment, concerns about an over-reliance on this particular format have been cited (7). Perhaps one of the most significant issues with the OSCE relates to the logistics inherent in its delivery due to the expansive resourcing involved, including personnel required for set up and delivery, as well as the time and cost required to run such an event (8). Further still, some critics have challenged the notion that OSCE testing confers a superiority in its psychometrics due to a lack of evidence that the this clinical assessment modality provides more rigorous assessment data than other assessment methods (9, 10). In recent times, the authenticity of the OSCE as an the assessment of clinical practice has been further controverted given that real patients are often absent from the actual assessment—and are instead represented by actors adhering to standard scripts (11).

With the advent of COVID-pandemic conditions in 2020, the degree of reliance on the OSCE as the main tool for clinical assessment became acutely problematic for many institutions of the health professions due to an exacerbation of logistical issues inherent in delivery. Due to the existence of physical distancing regulations, it was not consistently feasible to bring together the usual stakeholders (students, examiners, simulated patient, academic and administrative staff, and invigilators) in 2020 and 2021 to deliver the OSCE in its traditional, face-to-face modality. Nonetheless, ‘the show must go on', as it were—and, despite the disrupted environment, educators still required competency grading information to inform decision-making processes for student progression. As a consequence, health professions educators were challenged to design alternate assessment strategies to gather datapoints, including developing new assessment tools, or put greater emphasis on alternate, existent ones, to measure student clinical performances in the context of COVID-19-safe public health guidelines.

In response to pandemic restrictions, some institutions rapidly transformed from a face-to-face OSCE to a virtual OSCE (vOSCE) modality to allow for the assessment of clinical skills via remote learning platforms (12, 13). However, recent studies have shown that, although the vOSCE is adaptable to diverse examination formats, it is not deemed to be completely “fit for purpose” (14). Specifically, while the vOSCE may be effective for assessment of some clinical skills such as history-taking and communication skills, particularly in the context of a growing emphasis on Telehealth (15, 16), the ability to observe physical examination and procedural skills via virtual delivery is severely limited.

To assess student clinical competence during the pandemic environment, some educators relied more heavily on existing clinical assessment tools to the extent that they could be delivered, such as workplace-based assessments (WBAs), including the Mini-Clinical Evaluation Exercise (mini-CEX), Direct Observation of Procedural Skills (DOPS), Case-based discussions (CBD), and In-training assessment (ITA) (17, 18). Although WBAs provide for comprehensive and integrated assessment opportunities, they are typically used for formative purposes, and are considered to have limited utility in summative assessment due to their lack of standardization (19), despite the existence of evidence for the rigor and validity of the programmatic approach using WBAs (20). Nonetheless, during a time when health professions educators were bound by pandemic conditions, the use of WBAs allowed for summative assessment opportunities in circumstances where delivery of the traditional OSCE was not possible. In effect, by shifting away from the OSCE in favor of WBAs, health professions educators were able to capture multiple (albeit unstandardised) datapoints for student clinical competency grading during a pandemic-disrupted year.

As we now enter this new phase of the peri-pandemic world, there is no better time to review the traditional reliance on the OSCE to ensure that future clinical assessment strategies reflect best practice (11, 21). In elucidating the currency of the OSCE, we draw on experiences from changes in our context to examine the important question: Will health professions educators and other stakeholders accept the durability of pandemic-related adaptations made to clinical assessments, or will we simply return to the pre-COVID-19 OSCE-centric status quo? In light of lessons learned by Australian medical schools during the pandemic, this study explores the perceptions of clinical assessment stakeholders (examiners, students, simulated patients and administrators) in relation to the future role of the traditional OSCE.

## Methods

### Study Context

This study is part of a larger project that explored the experiences of all stakeholders hosting or attending virtual OSCEs (vOSCEs) at medical schools that are affiliated to the Australasian Collaboration for Clinical Assessment in Medicine (ACCLAiM). ACCLAiM is comprised of 15 medical schools across Australia and New Zealand and fosters a community of practice centered around quality in clinical assessment (22–24).

### Study Design

The study utilized a qualitative design and focus group discussions (FGDs) to facilitate discussion and interactions within a nurturing environment (25). This work was conducted under Permit H6833, granted by the James Cook University Human Research Ethics Committee.

### Participants

An email invitation was sent to lead ACCLAiM academics from five medical schools involved in vOSCEs during the 2020 study period. Individual participants were purposively sampled by invitation from the academic assessment leads at participating medical schools. Prospective participants from all stakeholder groups, including academics, simulated patients, professional staff and students, were invited by email.

### Data Collection

Participants' perceptions about the utility of the OSCE in relation to other forms of clinical assessment was explored through FGDs which were conducted between November and December 2020 using online video-conferencing programs. The focus groups were held no more than 4 weeks following the vOSCE, with most being held within a week. The FGDs were conducted by the first-named author (BMA) who is an experienced qualitative researcher. Another member of the research team (KJ) organized the FGDs and served as an observer to ensure appropriate data acquisition. The aim of the study was reiterated, and verbal consent was obtained from the participants before commencing the FGDs. To avert social desirability bias during the discussions, participants were assured of the researchers' adherence to confidentiality and anonymity protocols and that there were no right or wrong responses. Additionally, care was taken to ensure that all participants were engaged equally to foster balanced perspectives in each focus group. The discussions were based on semi-structured open-ended questions such as, “does the OSCE still have a place in clinical assessment?” and “is the OSCE a sufficient tool to assess competence or does it need to be replaced?”. The FGDs continued until data saturation was achieved as judged by no emerging new information (26). Each FGD lasted between 40 and 60 mins, and were audio recorded.

### Data Analysis

An external transcription service was engaged to transcribe the audio-recordings from the FGDs. To enhance the credibility and trustworthiness of the results, the data was thematically analyzed by three members (BMA, SS and CR) of the research team, who independently coded and identified the emerging themes. Collated data were coded using a line-by-line open coding process. Emerging themes were identified using a constant comparison process, as advocated by Corbin and Strauss (25). All discrepancies were resolved in a consensus meeting involving the whole research team. The identified themes are presented using illustrative quotes.

## Results

Five FGDs were held, involving 23 participants (17 females and 6 males) from five medical schools and representing a broad range of stakeholder groups, including 11 academics, 3 simulated patients, 7 professional staff and 2 students (Table 1).

**Table 1:** FGDs participant characteristics*.

**FGD**	**Participant characteristics**
**1**	2x FPr; 1x FAc/QA; 1x Fac/Ex; 2xMSt; 1xFSP
**2**	2xFPr; 1xMPr; 3xFEx
**3**	1xMEx; 2xFPr
**4**	1xMSP; 1xFSP
**5**	4xFAc; 1xMAc

* *F, Female; M, Male; Pr, professional staff member; Ac, Academic; QA, Quality assurance visitor; St, student; SP, Simulated patient; Ex, Examiner*.

Thematic analysis of the FG transcripts revealed four major themes in relation to participants' views about the future of the OSCE in relation to other clinical assessments post-pandemic. These themes are (a) enduring value of the OSCE; (b) OSCE tensions; (c) educational impact; and (d) importance of a program of assessment. Illustrative quotes are presented and affixed with participants' demographic profiles. For example, participant S3-P16-M-Ex refers to School 3, Participant 16, Male, Examiner.

### Enduring Value of the OSCE

All stakeholders commenced the FGDs by outlining the benefits of the OSCE as a clinical assessment tool. Participants particularly valued that the OSCE is conducted in an objective manner within a simulated environment; and that the inclusion of a variety of stations allows for assessment of a range of clinical skills that are marked in a consistent fashion by examiners who are trained to standardize their ratings of observed student performances.

“*The OSCE is [conducted in] a very objective and simulated environment to test certain part of their [students] skills. Right? So one station, you might be focusing on communication skills or breaking bad news. And another station, you're focusing on physical examination, another station you focus on clinical reasoning.”* ***S3-P16-M-Ex***

“*The OSCE is easier to ensure the exam is consistent, in the form of auditing and examiners' training, and they have very good instructions. And there is even a format on how to mark the students. So, I think that binds the examiners and ensure they are more consistent compared to other observation-based clinical examination. It's definitely very important more so in the final year, so I don't think you can completely replace the model of the OSCE.”* ***S1-P2-F-AC/QA***

Importantly, from the student perspective, the ability to standardize the examiner marking makes the OSCE a fair assessment tool. Furthermore, to capitalize on this benefit, having a multitude of stations, each with a different examiner, enhances the fairness of the marking process. Participants also felt that the ability of the OSCE to include quality assurance/control measures provides an oversight to the assessment which enhances its fairness in an ‘unparalleled' way when compared to other clinical assessment formats.

“*It [WBA] opens the door for a lot of unfairness in terms of assessments. I think the model is that the more control that the uni has over how it assesses the students, the better. And the oversight you can have in an in-person OSCE, I think is unparalleled.”* ***S1-P6-M-ST***

“*You'd also want to make sure that you had different people doing each of the cases to make it fair, so that they are not always examined by the same person. Because I think that increases the fairness of it.”* ***S1-P5-F-AC/EX***

### OSCE Tensions

Despite the OSCE benefits outlined by participants, they reported that it is a costly and high-pressure examination—with the stress not only impacting the students, but also staff given the extent of logistics that need to be addressed in real time.

“*Just a high stakes summative, high stress environment within half a day to do the OSCE. I'm not saying you need to … you could scrap OSCE…. You still need to run OSCE to maintain objective way of measurement, but you can always supplement with other things.”* ***S3-P16-M-Ex***

“*I think overall, the tensions on the day…because in a live OSCE there's just so many pieces that have got to all fit together so precisely, and the timing.”* ***S2-P8-F-Ex***

Additionally, for complex cases where real patients are required, the arrangement logistics are often difficult for educators, noting that eligible patients may have co-morbidities that prevent them from presenting and remaining safely at the examination site for the duration of the assessment. The absence of real patients in the OSCE has implications for its authenticity.

“*At the moment, we're not allowed to have patients over the age of 65 - especially if they've got, you know, any sort of comorbidity like blood pressure or something. And that made it difficult because part of an exit OSCE... You know, you're supposed to be assessing geriatric care or chronic care. And how do you do that with an SP unless they are young but old looking.”* ***S2-P12-F-Ex***

Furthermore, with the COVID pandemic, the traditional face-to-face OSCE had to be substituted with the virtual OSCE, limiting its ability to assess physical examination and procedural skills due to the COVID restrictions.

“*I think then we're saying that the limitations of the vOSCE are that we can perhaps only assess 50% of the range of skills that we need to assess for a graduating student. If we can't see their procedural skills, and their physical examination skills…We are talking about significant limitations of vOSCE as an exit exam.”* ***S2-P8-F-Ex***

### Educational Impact

In terms of educational impact, participants reported that the OSCE allows students to practice their skills within a safe learning environment.

“*And the traditional OSCE I think does that because I think it drives them [students] to get together in groups and [learn] in pairs, it drives them to see patients to practice their examination skills.”* ***S1-P5-F-AC/EX***

“*I think was okay in the sense that we have a formative OSCE first …so it is a safety net for the students as well.”* ***S1-P2-F-AC/QA***

However, the participants indicated that it isn't as effective as WBAs in driving student engagement and learning. They felt WBA provided authentic assessment opportunities which fostered better clinical practice.

“*In terms of workplace-based assessment [in comparison to OSCEs], I think it's very crucial, as S1-P5-F-AC/EX said, it increases student engagement with patients, it actually gets them to do things that doctors would normally do and actually be prepared to be interns.”* ***S1-P6-M-ST***

Participants also felt that in comparison to the OSCE, the WBA utilizes a more holistic approach to ensuring students' work-readiness as it focuses on professional behaviors and identity, though the challenge with this assessment format is the more subjective marking rubric. Nonetheless, the participants emphasized that both the OSCE and the WBA have their unique places as major clinical assessment formats and shouldn't be seen as competing against each other.

“*Yeah, I think I agree with continuous workplace-based assessment, that will be useful. I think the major challenge is to have a good marking rubric, that could be easily applied to whatever workplace competency that you are going to measure. So that actually complements the OSCE, I would say. It can't replace the whole OSCE because, as you know, OSCE is very objective and [provides] a simulated environment to test certain part of their skills. Right? You can't replace OSCE but at least you can take away part of the assessment marks into a more continuous assessment. So, I think workplace-based assessment is for more like a 360 measurement of how the student performs in different ways. So, you can look at the professionalism, you can look at how they interact with the patient, how, what their clinical knowledge are, interact within the team, right. So, there are different ways that you measure in a workplace-based format assessment. So, I will have it as a different assessment modality, rather than competing against each other.”* ***S3-P16-M-Ex***

“*I guess in terms of assessments in any assessment, the goal of it at the end of the day is to see whether we'll be good doctors. And in terms of a hierarchy, workplace-based assessments are obviously number one, because it's literally us practicing to be doctors. Next, come OSCEs, and next comes written exams. And the biggest problem kind of working up and down the ladder is that although the top of the ladder is most resemblant of what we actually need to be doing and good at, it's also the most difficult to standardize. Whereas at the very bottom, a multiple-choice exam is the easiest thing to standardize.”* ***S1-P7-M-St***

### Importance of a Program of Assessment

Participants acknowledged that not all cases are assessable with an OSCE and therefore endorsed the utilization of a program of assessment that allows for the inclusion of different assessment modalities to obtain a whole picture of students' clinical competence.

“*So, it could be in terms of, as we're saying, with programmatic stuff [assessment]. I love S4-P21-F-QA's idea about, you could have some standardized assessments in later years, you could have some vOSCE, you could have some workplace-based assessments. And the really big rigorous OSCE may be of much more use in the junior years to say they are to standard or they're not.”* ***S3-P20-F-QA***

In terms of adopting a programmatic assessment approach, participants emphasized the need for a mixture of assessment methods with learner performance been monitored longitudinally, instead of single point of high stakes assessment.

“*I don't know what the answers are. But I think perhaps we do need to get away from thinking of an OSCE as something that happens on this day and they do X number of stations.”* ***S4-P21-F-QA***

## Discussion

As a high-fidelity rapid sampling tool, the OSCE has become ascendant in clinical assessment due to its ability to deliver robust and defensible quantification of candidates' performances. As this study recognizes from multiple stakeholder perspectives, the ability of the OSCE to deliver an objective and standardized assessment, where there is spread of content sampled and ability to provide central quality control, are essential elements. These assessment qualities confer an enduring value to the OSCE that will ensure it continues to play a significant role in clinical assessment.

Nonetheless, this study highlights that the logistical tensions associated with OSCE delivery cannot be overlooked. Medical educators are currently experiencing a general “turning point” in the way they design clinical assessment on the back of rapid changes made during the pandemic, with reflective practice driving the scrutiny of previous approaches. Since the advent of COVID-19, novel assessment modalities and extant, but less rigorous, formats have been engaged to provide medical schools with competency grading information that would have previously been predominantly derived from a large-scale OSCE. In light of new learnings gleaned from clinical assessment experiences over the past 2 years, our study findings reflect the discourse occurring in medical schools as to how to retain all that is desirable about the OSCE (rigor, standardization, and direct observation from multiple sources) alongside a balance of assessment points that are authentic, patient-centered and situated in the clinical workplace.

In terms of educational impact, WBAs have been lauded for encouraging authentic clinical learning in real workplace settings, while the OSCE has been increasingly portrayed as encouraging “faux” simulation, where candidates participate in robotic and superficial interactions devoid of the intricacies and intimacies of team-based patient care (11). Although OSCE criticism expresses valid concerns regarding how a poorly designed OSCE may drive candidates to disengage from learning authentic medical practice, station design improvements and the eliciting of patient judgements can address some of these concerns (27, 28). Furthermore, the ability to integrate quality assurance measures into the OSCE process is significant, and clinical assessment communities of practice have been established to guide OSCE quality improvements as a fundamental outcome of their activities (23, 24).

Future programs of assessment may consider at what point in the course the OSCE is used, in what form and for what purpose. Simulation in medical education has the significant and recognized advantage of providing learners with standardized and challenging learning experiences while preserving patient safety (29). Certain content e.g. recognizing the deteriorating patient, is specifically suited to simulated teaching and assessment methods. In an OSCE, candidates can be assessed in critical care competencies to ensure attainment of a minimal acceptable level of learning and thus avoid exposing patients to the danger of below standard performances. In contrast, WBA provides the ability to observe the candidate in an authentic setting, with framework tools, such as graduate competencies and Entrustable Professional Activities (30), providing a structured method of sampling and measuring *in situ* performances.

This study highlights that, while it seems the OSCE is here to stay, rather than standing alone, a programmatic assessment approach may be preferable. Programmatic assessment allows for comprehensive information gathering about the leaner's competence, involving the culmination of information from a variety of assessment instruments to further guide the learner, and for high stakes decision-making by educators regarding suitability for student progression (and graduation) (31). As suggested by this study, the use of the OSCE and WBAs are not seen as mutually exclusive assessment strategies; and in fact, our participants felt they could be used in a complementary fashion to maximize attainment of competency grading information.

This study draws on the perspectives of multiple diverse stakeholders from 5 institutions in the Australian context during the COVID-19 pandemic disruption to medical education and assessment. Globally, the experience of pandemic disruption has not been uniform, and it is anticipated that exploring international perspectives on the future of the OSCE would yield additional insights. Furthermore, as the focus group discussions focused on the use of OSCE and WBAs only, it would have been impactful to seek a wider perspective about the use of a programmatic assessment approach, and how (and when) each assessment modality fits into this model.

## Strengths and Limitations

The major strength of this study is use of an exploratory study to interrogate the perceptions of clinical assessment stakeholders (examiners, students, simulated patients and administrators) in relation to the future role of the traditional OSCE, which extends the literature on this topic. However, the study consisted of participants from five different medical schools across four key stakeholder groups, with variable engagement between these groups, which may impact on the transferability of the study findings to other settings. The reduced student and simulated patient participation may have been a result of timing; these FGDs were held after the exam period where many prospective participants were no longer available. This may also have been hampered by the online nature of the FGDs. It is likely that a face-to-face format would have enhanced recruitment of participants. However, the online format facilitated FGDs across Australia during a time when pandemic conditions had restricted travel.

## Conclusion

Our findings suggest that the OSCE has survived the pandemic and may retain a place in clinical assessments due to its perceived fairness, standardization and ability to yield robust results. However, new experiences and reflective practice have driven a re-imagining of its role alongside WBAs in the new peri-pandemic programs of assessment.

## Data Availability Statement

The original contributions presented in the study are included in the article/supplementary material, further inquiries can be directed to the corresponding author/s.

## Ethics Statement

The studies involving human participants were reviewed and approved by James Cook University Human Research Ethics Committee. This work was conducted under Permit H6833. The patients/participants provided their written informed consent to participate in this study.

## Author Contributions

BM-A conceived the study. KJ facilitated data collection. BM-A conducted the focus groups. BM-A, SS, and CR conducted data analysis. All authors contributed to interpretation of results, writing the original draft of the manuscript, reviewed, edited, and accepted the final version of the manuscript.

## Conflict of Interest

The authors declare that the research was conducted in the absence of any commercial or financial relationships that could be construed as a potential conflict of interest.

## Publisher's Note

All claims expressed in this article are solely those of the authors and do not necessarily represent those of their affiliated organizations, or those of the publisher, the editors and the reviewers. Any product that may be evaluated in this article, or claim that may be made by its manufacturer, is not guaranteed or endorsed by the publisher.
